# *Saccharomyces cerevisiae *glycerol/H^+ ^symporter Stl1p is essential for cold/near-freeze and freeze stress adaptation. A simple recipe with high biotechnological potential is given

**DOI:** 10.1186/1475-2859-9-82

**Published:** 2010-11-03

**Authors:** Joana Tulha, Ana Lima, Cândida Lucas, Célia Ferreira

**Affiliations:** 1 CBMA (Centre of Molecular and Environmental Biology), Department of Biology, University of Minho, Campus de Gualtar, 4710-057 Braga, Portugal

## Abstract

**Background:**

Freezing is an increasingly important means of preservation and storage of microbial strains used for many types of industrial applications including food processing. However, the yeast mechanisms of tolerance and sensitivity to freeze or near-freeze stress are still poorly understood. More knowledge on this regard would improve their biotechnological potential. Glycerol, in particular intracellular glycerol, has been assigned as a cryoprotectant, also important for cold/near-freeze stress adaptation. The *S. cerevisiae *glycerol active transporter Stl1p plays an important role on the fast accumulation of glycerol. This gene is expressed under gluconeogenic conditions, under osmotic shock and stress, as well as under high temperatures.

**Results:**

We found that cells grown on *STL1 *induction medium (YPGE) and subjected to cold/near-freeze stress, displayed an extremely high expression of this gene, also visible at glycerol/H^+ ^symporter activity level. Under the same conditions, the strains harbouring this transporter accumulated more than 400 mM glycerol, whereas the glycerol/H^+ ^symporter mutant presented less than 1 mM. Consistently, the strains able to accumulate glycerol survive 25-50% more than the *stl1Δ *mutant.

**Conclusions:**

In this work, we report the contribution of the glycerol/H^+ ^symporter Stl1p for the accumulation and maintenance of glycerol intracellular levels, and consequently cell survival at cold/near-freeze and freeze temperatures. These findings have a high biotechnological impact, as they show that any *S. cerevisiae *strain already in use can become more resistant to cold/freeze-thaw stress just by simply adding glycerol to the broth. The combination of low temperatures with extracellular glycerol will induce the transporter Stl1p. This solution avoids the use of transgenic strains, in particular in food industry.

## Background

Preservation by low temperatures is widely accepted as a suitable method for long-term storage of various types of cells. Freezing has become an important means of preservation and storage of strains used for many types of industrial and food processing, including the production of wine, cheese and bread. In particular, frozen dough technology is extensively used in the baking industry, one of the largest in the world due to the central role of bread as a dietary product. In spite of its commercial relevance, yeast mechanisms of tolerance and sensitivity to freeze or near-freeze stress are still poorly understood.

Cold, near-freeze and freeze-thaw stress cause various types of damage to the cells, mainly due to the formation of intracellular ice crystals and dehydration during the freezing process, including effects upon the structure of the cell wall, the membrane, and the cellular organelles. Cryoprotectants are largely used to prevent some of these events. They promote the excretion of water, decreasing the formation of ice crystals. Me2SO and trehalose are well-established cryoprotectants, while certain amino acids, such as proline, arginine and glutamate, have also demonstrated a significant cryoprotective effect in *S. cerevisiae *[1]. The use of Me2SO in food preparation is not possible due to its toxicity; on the other hand the mechanisms of action of trehalose are still not fully elucidated. A recent work [2] showed that cell viability after freezing/thawing process increased by supplementing the broth with copper ions, suggesting that insufficiency of copper ion homeostasis may be one of the causes of freeze-thaw injury. However, these ions toxicity does not allow their easy incorporation in food products. Finally, glycerol is also a powerful cryoprotectant, similarly to trehalose, for many types of cells including *S. cerevisiae *[3]. Since glycerol is chemically inert and presents biological negligible toxicity, it is extensively used in a broad spectrum of applications, from pharmaceutical adjuvant or daily care products additive, to the preservation of cells and enzymes at extremely low temperatures [3].

Unlike with other stress agents it is not consensual that the exposure of cells to freeze/thaw conditions may lead to improvement of tolerance. Park and co-authors [4] described that unlike other eukaryotes *S. cerevisiae *did not display adaptation to freeze/thaw stress, neither following repeated freeze-thaw treatments, nor following pre-treatment by cold shock. Yet, in the same work the authors showed that cross protection between freeze/thaw stress and a limited number of other types of stresses existed. Namely, freeze/thaw tolerance could be induced by pre-treatment with H_2_O_2_, cycloheximide, mild heat shock, or by NaCl. Consistently, recent studies described the yeast adaptation to freeze/thaw stress by combination of UV mutagenesis with 200 rounds of freezing/thawing [5] or by pre-growth at 15°C [6]. Another study [7] showed that below 10°C, yeast have an adaptive response that protects viability to subsequent exposure to low or freezing temperatures. More recently it was shown that cells of industrial strains growing at 15°C displayed enhanced freeze and frozen-storage resistance than those grown at 30°C [8].

The adaptation of yeast cells to low temperatures implies a change in genes expression [2] with consequences at the level of metabolism [3,4], membrane physio-chemical properties [8] and expectedly the production and accumulation of trehalose [1,9] and/or glycerol [10,11]. In this regard, two engineering approaches were performed in order to increase the intracellular glycerol accumulation in baker's yeast [10,11], being the most promising genetic modification, the deletion of *FPS1 *encoding the yeast glycerol channel [11]. These engineered cells showed an increase in intracellular glycerol accumulation at 30°C [11], accompanied by higher survival after 7 days at -20°C. Distinct studies reported a close correlation between the intracellular glycerol level and the fermentation ability [12] as well as other benefits for the shelf life of soaked yeast products and for the leavening activity [13].

The *S. cerevisiae *glycerol active transporter, Stl1p, is expressed under gluconeogenic conditions, as well as under osmotic shock and stress, and is repressed by glucose [14-17]. In induction conditions, it plays an important role for the fast accumulation of glycerol [14-17]. In a previous work, our group found that *STL1 *is also highly expressed at 37°C, and that this over-expression is fully accompanied by a hyper activity of Stl1p, meaning an elevated glycerol active uptake velocity [16]. Furthermore, we also described that under these conditions the repression by glucose was alleviated [16].

Despite the numerous efforts, up to date, there is still no industrial strain with appropriated high tolerance to cold, near-freeze and freeze stress. In that regard, our work contributes significantly to achieve this long pursued aim, by circumventing the need for new genetically improved strains with a simple broth manipulation. This is obtained through the contribution of the inducible activity of the yeast glycerol symporter, Stl1p, for the accumulation and maintenance of high glycerol intracellular levels at cold and near-freeze stress. These high levels naturally allow cells to tolerate temperature down-shifts.

## Results

### Stl1p is essential for cold/near-freeze and freezing tolerance

Several glycerol-related mutants were previously shown to be sensitive to low and freeze-temperatures. These include *ara1Δgcy1Δgre3Δypr1*, *Δgpd1Δgpd2Δ*, and *fps1Δ *[10,11]. Moreover, intracellular accumulation of glycerol has shown to be important for low and freeze-stress tolerance [6,10,11]. This led us to check survival of the mutant defective on the glycerol/H^+ ^symporter Stl1p, as well as the *gpdΔgpd2Δ *mutant, at low (4°C) and freeze-thaw (-20°C) temperatures. These assays were performed in repression (YPD) and derepression (YPGE) conditions.

**Figure 1:**
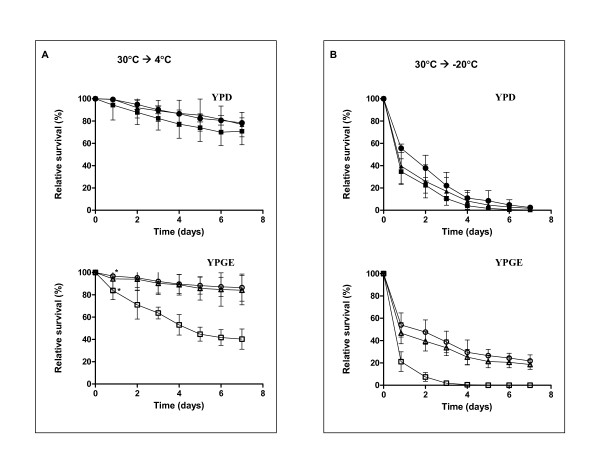
**Stl1p is essential for cold/near-freeze and freezing tolerance**. Relative survival (%) of the wt (circle) and the mutants defective on glycerol permease, *stl1pΔ *(square) and defective on the isoenzymes from glycerol-3P dehydrogenase *gpd1Δgpd2Δ *(triangle), was determined in YPD (close symbols) and YPGE (open symbols) medium, for cells kept at 4°C (A) or -20°C (B). * This 1^st ^time point corresponds to 20 h incubation which was used for transport assays.

When incubated at 4°C, the strain defective on glycerol symporter, Stl1p, displayed a poor survival when compared with wt or *gpd1Δgpd2Δ *(Figure 1A). This was more pronounced in YPGE than on YPD. Conversely, the strains harbouring the transporter, wt and *gpd1Δgpd2Δ*, survived better in glycerol containing media, YPGE (Figure 1A, open symbols). Since incubation at -20°C and thawing create a more stressful condition to the cells, generally speaking, a decreased survival was expected. Indeed, that was observed, and again *stl1Δ *mutant survived less than wt or *gpd1Δgpd2Δ *(Figure 1B). Again, this was more pronounced in YPGE than in YPD. The mutant was almost totally dead on the fourth day (0.5% survival) whereas the wt and *gpd1Δgpd2Δ*strains still displayed a relative survival of around 24%. Once more, the latter strains survived better in YPGE than in YPD.

### Stl1p is induced by low temperatures

**Figure 2:**
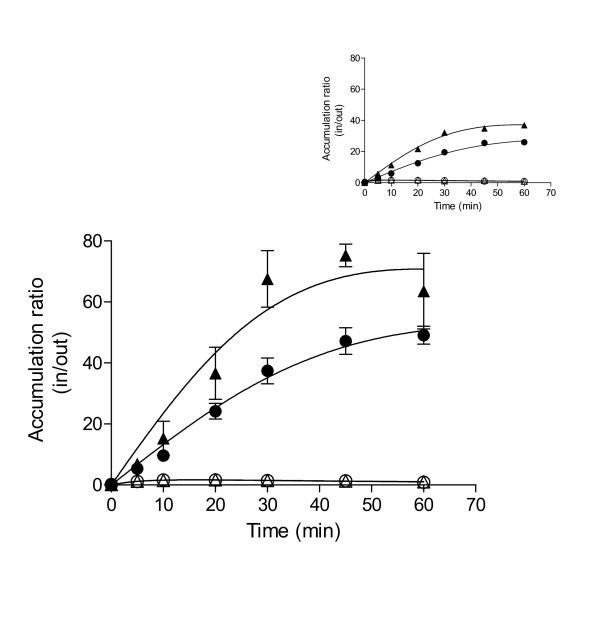
**Activity of the glycerol/H^+ ^symporter under cold/near-freeze stress**. Accumulation ratios of [^14^C]-glycerol on exponential growing cells on YPGE subjected to incubation at 4°C during 20 h: wt cells (triangle) and *gpd1Δgpd2Δ *mutant cells (circle). Cells were assayed in the absence (close symbols) or in the presence (open symbols) of CCCP. In the insert are represented the control experiments of non-stressed cells.

In order to evaluate glycerol transport at low and near-freeze temperatures, wt cells were grown on induction conditions (30°C, YPGE) and kept at 4°C for 20 h to transport assays (Figure 1*). Transport assays were performed as usual, *i.e*. at 30C, allowing the cells to adapt for a brief period to this high temperature. We observed that the accumulation ratio exceeded the equilibrium, reaching ≈ 75 times in/out, and that was sensitive to CCCP (Figure 2), whereas in with the control cells (not submitted to the 4°C incubation), the maximum in/out ratio was just ≈ 36 (Figure 2 insert), suggesting less amount of protein.

Identical assays were done with the *gpd1Δgpd2Δ *mutant, leading to equivalent results (Figure 2 and 2 insert), except that the maximums ratios attained were respectively, ≈ 49 and ≈ 26 times in/out. Furthermore, Fps1-mediated diffusion rates [18] were measured and maintained constant in all conditions and strains (not shown).

**Figure 3:**
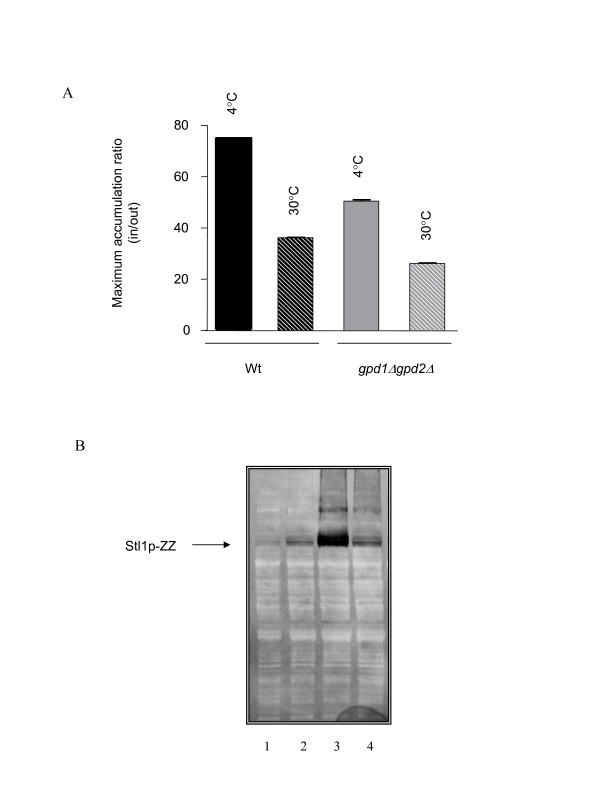
**Induction of active glycerol transport**. (A) Cells of wt and *gpd1Δgpd2Δ *mutant were grown to exponential phase on YPGE at 30°C (stripped bars) and subjected to incubation during 20 h at 4°C (full bars). (B) The same batches of cells were used to detect the levels of Stl1p expression by Western Blot using *STL1-ZZ *construction: 1 and 4 - *gpd1Δgpd2Δ *cells at 30°C and 4°C respectively; 2 and 3 - wt cells at 30°C and 4°C respectively.

The same batches of cells used to measure glycerol accumulation were analyzed by Western Blot for Stl1p as described before [16]. A significantly higher amount of Stl1p was detected in wt cells cultivated on YPGE and submitted to 4°C incubation, when compared with the one detected in same cells not subjected to such treatment (Figure 3A). Identical result was obtained in the *gpd1Δgpd2Δ *mutant although protein levels were smaller (Figure 3A). As expected, the levels of Stl1p and the glycerol uptake activity maximum accumulation correlate directly (Figure 3A and 3B).

### Glucose repression over Stl1p is not overcome by low temperatures

In a previous work [16], we showed that the glucose repression over *STL1 *expression [14,15,17] was overcome at 37°C. To verify whether this could be happens also at low temperatures, wt cells were grown on glucose based medium (YPD), subjected to an incubation at 4°C during 20 h and the glycerol transport-derived accumulation measured. We found that, in opposition to what happens at 37°C but in agreement with the regulation by glucose of this system at standard temperatures, accumulation did not exceeded equilibrium, and was not sensitive to CCCP (not shown), suggesting *STL1 *was not induced. Additionally, *gpd1Δgpd2Δ *mutant yielded identical results (not shown). As before, Fps1-mediated diffusion rates were measured and subsisted constant for the three strains (not shown).

Again, the same batches of cells used to measure glycerol accumulation were analyzed by Western Blot for Stl1p, and in YPD, regardless of cells (both, wt and mutant) being incubated or not, at 4°C, glucose repression was active; therefore no protein was found (not shown).

### Intracellular glycerol accumulation is enhanced at low temperatures with the contribution of Stl1p

Intracellular glycerol concentration was determined by HPLC [19] in the same batches of cells used to measure glycerol accumulation as well as to analyze Stl1p expression by Western Blot. Additionally, the *stl1Δ *mutant was also analysed. Considerable high content of intracellular glycerol was found in the wt cells cultivated on YPGE (*STL1 *induction conditions), and submitted to cold/near-freeze stress, in comparison to the amount observed in the cells not stressed (Table 1). In the mutant unable to produce regular amounts of glycerol, *gpd1Δgpd2Δ*, the same pattern was observed (Table 1). Regarding wt and *gpd1Δgpd2Δ *cells cultivated on YPD and submitted to the 4°C incubation, residual amounts of intracellular glycerol were observed (Table 1), similar to the values previously determined in YPD alone [20]. On the other hand, and in clear contrast, the *stl1Δ *mutant presents only trace amounts of intracellular glycerol on YPGE while on YPD at 4°C it behaves equally to the two other strains (Table 1).

**Table 1:** Intracellular glycerol contents (mM).

	Experimental procedures			
	
Cell type	YPGE, 20 h 4°C	YPGE, 30°C	YPD, 20 h 4°C	YPD, 30°C
Wt	453.8 ± 0.011	153.0 ± 0.021	1.190 ± 0.016	0.750 ± 0.35^∂^
*stl1Δ*	0.196 ± 0.019	0.167 ± 0.014	1.205 ± 0.011	1.091 ± 0.012
*gpd1Δgpd2Δ*	427.1 ± 0.020	138.8 ± 0.023	0.981 ± 0.013	1.1 ± 0.021^∂^

^∂ ^Results on YPD at 30°C were obtained before (Oliveira and Lucas, 2004) and yielded residual amounts on wt and *gpd1Δgpd2Δ*..

## Discussion

We demonstrated here that disruption of the glycerol/H^+ ^symporter gene *STL1 *led to a pronounced decrease in the levels of intracellular glycerol, with concomitant diminished survival to cold/freeze-stress. These results clearly suggest an important role of Stl1p to maintain the intracellular glycerol contents and ultimately in the resistance to cold/near-freeze-stress.

*STL1 *induction by osmotic shock is regulated by the High Osmolarity Glycerol (HOG) pathway [15,16,21]. Is a fact that, several other stimuli, besides hyper-osmolarity, activate the HOG pathway, namely, heat stress, oxidative stress, changes in turgor pressure and, more recently cold and freeze stress had been added to the list [6,22,23]. Curiously, hyper-osmotic stress and cold/near freeze and freeze stress share some common physiological features. The most limiting factor cells have to cope, in these conditions, is the low water activity (a_w_). At low/near-freeze and freeze temperatures occurs a re-organization on the assembly of the water molecules, which lean to crystallize, leading in turn to the reduction of its accessibility for the cells. The same takes place with hyper-osmotic stress though, through a different mechanism [24]. Furthermore, both stresses are known to reduce the membrane fluidity [22,25,26]. It is therefore possible that, the induction of *STL1 *by cold/near freeze and freeze stress happens via the Hot1p/Hog1p pathway regulation. Further work must be done to clarify this hypothesis.

Our group, had reported in a previous work [16] an important role of Stl1p in the response of *S. cerevisiae *to high temperatures stress, having proposed that the glycerol/H^+ ^symporter contributes to the fine tuning of glycerol internal levels not only on such condition as to other physiological conditions. Herein, we report cold/near-freeze/freeze stress as other conditions to add to the list, which include already the established diauxic phase transition, heat-stress and osmotic-stress. In the same work, it was stated that the glucose repression, to which glycerol/H^+ ^symporter is subjected [15-17], was alleviated by high temperatures, both at the level of protein expression and activity, which implied proper traffic and localization besides protein synthesis [16]. At the date, as now, that was the unique report of such phenomenon. In the present work, we show that under cold/near-freeze stress the glycerol uptake symporter is still under glucose repression. This result reinforces the already suggested idea that even sharing some features, heat-stress response and cold/near-freeze response are different processes [7].

*S. cerevisiae *cells growing in glucose based medium are known to produce glycerol [20,27], from which a considerable amount is excreted into the medium, through the glycerol facilitator channel Fps1p [11,18,20,27]. It has been demonstrated that under osmotic-salt stress Fps1p channel closes, avoiding excretion into the medium [28,29]. And, despite of the recognized relevance of glycerol as thermoprotectant both at high and low temperatures, the fact is that, under temperature stress any study has verified the truly dynamics of Fps1p channel or even the intracellular glycerol content. Izawa and co-author's [11] showed that the *fps1Δ *mutant was more resistant to freeze stress (up to 7 days at -20°C) than wt. Indirectly, this probably means that the channel doesn't remain closed, at least not all the time. We found high levels of intracellular glycerol content in wt and *gpd1Δgpd2Δ *mutant cells subject to cold/near-freeze/freeze stress, which were not found in the cells lacking the transporter, *stl1pΔ *mutant. In accordance wt and *gpd1Δgpd2Δ *mutant strain survive much better than *stl1pΔ *mutant both at 4°C and -20°C. Moreover, the intracellular glycerol contents found in these conditions are quite high, comparing with the ones determined under osmotic stress [20]. Taking all these studies in consideration, we propose that the contribution of the glycerol/H^+ ^symporter Stl1p for the accumulation of intracellular glycerol and consequent improved tolerance to cold/near-freeze/freeze stress, is crucial.

## Conclusions

The combination of low temperatures with extracellular glycerol highly induces the permease Stl1p, promoting an increase in the glycerol intracellular levels which translates into improved cell survival. Based on these results, and in a biotechnological perspective, any *S. cerevisiae *wt strain already in use can be converted into a more resistant strain to freeze and near-freeze stress and therefore become even more interesting for industrial uses, just by simply adding glycerol to the broth. Glycerol is a considerable low cost raw material from many fermenting industrial processes, mainly biodiesel production. This solution avoids the use of transgenic strains in particular in the food industry.

## Methods

### Strains, media and growth conditions

*S. cerevisiae *strains used in this work were FVVY24 [15] referred ahead as *stl1Δ *mutant, FVVY28 [15] referred ahead as wt, and FVVY39 [15] referred ahead as *gpd1Δgpd2Δ *double mutant. Batch cultures of yeast were performed aerobically at 30°C and 200 rpm, unless differently stated. Growth complex medium (YP: 1% (w/v) yeast extract; 2% (w/v) peptone) was supplemented with 2% (w/v) glucose (YPD) or 1% (w/v) ethanol combined with 1% (w/v) glycerol (YPGE) as carbon and energy sources.

### Cold/near-freeze and freezing tolerance test

Cells were cultured in YPD medium at 30°C and collected during exponential phase, washed once and diluted with sterile water at room temperature to an optical density (OD) of 1.0 at 600 nm (approximately 1 × 10^6 ^cells/ml) and incubated at 4°C or -20°C. Cell survival was monitored as follows: at each time point tubes were removed from storage incubators, allowed to defrost under established conditions, *i.e*. 30°C for 10 min [2,8]. Ten fold serial dilutions were performed in 1.5 ml sample tubes, from which 10 μl were spotted five times on YPD and YPGE plates. Colonies were scored after 48 h incubation at 30°C.

### Glycerol transport studies

The assays on proton symporter activity-driven accumulation were performed as described before [17,18,30]. The *S. cerevisiae *intracellular volume used to calculate the intracellular glycerol concentrations was determined previously [30].

### Western Blotting

The assay was performed as before [15]. Cells (0.2 mg dry weight) were collected by centrifugation, and protein extracts were prepared as previously [15]. The total amount of proteins present in each strain protein extract was measure by Bradford method. Identical amounts of protein controlled by this method were used to load two separate SDS-PAGE (10%) gels. One of the SDS-gels was stained with Coomassie Brilliant blue and the other proceeded for transference to the nitrocellulose membrane. The efficiency of this transference was checked by staining the membrane with Ponceau solution. The membranes were then incubated with a commercial mixture of horseradish peroxidase and antiperoxidase rabbit antibody (PAP, cat. no. Z0113, Dako-Cytomation A/S, Copenhagen, Denmark). The reacting polypeptides were visualized using ECL Plus Western Blotting Detection System (Amersham Biosciences) and an Image Analysis System ChemiDoc XRS (Bio-Rad, Laboratories Inc.) with Quantity-One 4.5.0 Software (Bio-Rad, Laboratories Inc.).

### Measurement of intracellular glycerol content

Identification and quantification of intracellular glycerol were performed by liquid chromatography (HPLC), using the same methodology and apparatus used before [19].

## Competing interests

The authors declare that they have no competing interests.

## Authors' contributions

JT and AL carried out the experimental studies, having contributed 60% and 40% respectively. CF conceived the project, co-supervised JT and AL, checked the data and wrote the manuscript. CL provided founds for the experimental studies execution and revised the manuscript. All authors read and approved the final manuscript.
